# School health systems under strain: an example of COVID-19 experiences & burnout among school health staff in Pima County, Arizona

**DOI:** 10.1186/s12889-023-16532-8

**Published:** 2023-08-25

**Authors:** Amanda M. Wilson, Priyanka Ravi, Nicole T. Pargas, Lynn B. Gerald, Ashley A. Lowe

**Affiliations:** 1 Department of Community, Environment & Policy, Mel & Enid Zuckerman College of Public Health, University of Arizona, 1295 N. Martin Ave. A233, Tucson, AZ 85721 USA; 2 Department of Health Promotion Sciences, Mel & Enid Zuckerman College of Public Health, University of Arizona, Tucson, AZ USA; 3 Health Services Department, Marana Unified School District, Marana, AZ USA; 4 Office of Population Health Sciences in the Office of the Vice Chancellor for Health Affairs, University of Illinois Chicago, Chicago, IL USA; 5 Asthma & Airway Disease Research Center, University of Arizona Health Sciences, Tucson, AZ USA

**Keywords:** School health staff, COVID-19, Pandemic, Challenges, Experiences, Qualitative study, Focus group, Interviews

## Abstract

**Background:**

School health staff lead and provide a variety of care for children in schools. As school districts have navigated the COVID-19 pandemic, school health staff have faced unprecedented challenges in protecting the health of students and school staff. Our objective was to qualitatively characterize these pandemic challenges and experiences of school health staff in Pima County, Arizona to identify gaps in school health staff support for improving future emergency preparedness.

**Methods:**

We conducted two focus group discussions (FGDs) with 48 school health staff in Pima County, Arizona in two school districts using a discussion guide including ten open-ended questions. The FGDs were audio recorded and transcribed verbatim. We used the socioecological model (SEM) to organize the thematic analysis and generate codes and themes; data were analyzed using Atlas.ti software.

**Findings:**

The pandemic has significantly challenged school health staff with new pandemic-related job tasks: managing isolation, vaccination, and developing/implementing new and evolving COVID-19 guidelines. School health staff also reported increased stress related to interactions with parents and school administration as well as frustrations with rapid changes to guidance from the health department and policy makers. A common issue was not having enough staff or resources to complete regular job responsibilities, such as providing care for students with non-COVID-19 related health issues.

**Conclusions:**

Increased workload for school health staff resulted in physical burnout, mental distress, and disruption of core functions with long term implications for children’s health. These focus groups highlight the need for improved emergency preparedness in schools during pandemics or infectious disease outbreaks. These include basic infrastructure changes (e.g., personnel support from health departments for tasks such as contact tracing to enable school nurses to continue core functions), and increased funding to allow for hazard pay and more school health personnel during emergency situations. In addition, basic school health infrastructure is lacking, and we should include a licensed school health nurse in every school.

**Supplementary Information:**

The online version contains supplementary material available at 10.1186/s12889-023-16532-8.

## Introduction

School health staff are primary health care professionals who lead health services in schools and practice in a holistic manner to address the needs of schoolchildren and school personnel [1]. Some of their core duties include screening and early detection of impairment of hearing [2] and vision [3], and conduction of immunizations [4]. They serve a vital role in addressing students’ health problems by being involved in school based asthma care [5], diabetic care [6], management and referral for Ear, Nose, and Throat (ENT) infections [7], facilitating dental screening, assessing children’s immunization status per requirements of U.S. states, and managing acute and chronic chare [8–10]. They also ensure positive school experiences for students by providing support for students with mental health disorders [11]. In addition, they often serve as a health resource for school personnel. School health staff teams may be composed of registered professional school nurses, licensed practical nurses/licensed vocational nurses (LPN/LVN), and unlicensed assistive personnel (UAPs) [10].

While the American Academy of Pediatrics suggested schools have at least one professional school nurse and most advocate for a full-time licensed nurse [12], even before the COVID-19 pandemic, many schools had none [13, 14]. There was already pressure on the school health system prior to COVID-19, and the pandemic exacerbated staffing challenges with new occupational duties, risks, burdens, and stressors on a global scale [1, 15–17].

The effect of COVID-19 on the mental health of school health professionals has been demonstrated internationally. Even despite cultural, geographical, and political differences across international settings, there are common findings related to increased strain and mental health burdens on school health professionals. In a study in Sweden, Martinsson et al. (2021) reported that the impact of policies and decisions on global and local levels affected the work situations of school nurses as well as the school nurses' social, cultural, and professional experience. Another qualitative study on the COVID-19 experiences of school nurses in Hong Kong elucidated three major themes: “managing stress," "navigating the school through the pandemic," and "raising the profile of the school nurse professional” [1]. A mixed methods study conducted among school nurses in Hawaii [17], demonstrated the chronic negative emotions related to the pandemic, but also resilience and positive coping techniques. The full impact of COVID-19 on school health staff and on school health systems continues to be elucidated, and differences by community are important to capture to inform emergency preparedness efforts in preparation for future pandemics or outbreaks. A deeper understanding of not only COVID-19’s mental health impacts on school health professionals individually but also on interpersonal, organizational, community, and societal levels with which they interact and serve is needed.

One geographical area that requires more research is that of the U.S. Southwest, specifically Arizona, which had the highest rate of COVID-19 transmission in the world in early 2021 [18]. A recent post-pandemic survey conducted by the Centers for Disease Control and Prevention (CDC) indicated that the highest prevalence of poor mental health outcomes among school nurses was in Region 9, which includes the state of Arizona. School nurses in Region 9 reported the highest prevalence of symptoms of depression, anxiety, and posttraumatic stress disorder (PTSD) [19]. In contrast, a recent Arizona school health staff survey indicated that 50.9% of school health staff reported being “very involved” in COVID-19 planning efforts, and only 54.9% reported being “very prepared” for the pandemic [20]. When reporting the source that was the most helpful in addressing the pandemic, 36.3% selected county health departments while only 11.9% selected the CDC [20]. However, the results of this survey only provide quantitative information and do not contextualize school health professionals’ experiences. More qualitative data are needed to elucidate specific gaps in school health staff support during the pandemic so the state can better prepare for future pandemics and outbreaks. The objective of this study was to qualitatively explore the challenges and experiences of school health staff during the pandemic in Pima County, Arizona.

## Methods

### Ethical approval

Ethical approval was obtained from the Institutional Review Board at the University of Arizona (IRB number STUDY00000326). Permission to conduct the study was obtained from each school district administration. Oral consent was read aloud before the focus group discussions (FGDs), and participants were given the option to decline participation. Participants who declined were not included in the study; only participants who provided informed consent were included.

### Study participants & recruitment

Participants were recruited from January to February 2022 from two large public-school districts in Tucson, Arizona: one district with 12,400 students and 17 schools (10 elementary, 2 K-8, 2 middle and 3 high schools) and another with 14,000 students and 24 schools (8 elementary, 3 K-8, 1 K-12, 5 middle and 7 high schools). Focus groups were also conducted in this timeframe. The two school districts were chosen as a convenience sample based on their strong relationships with the research personnel, increasing the feasibility of conducting the focus groups during COVID-19. The school health staff included licensed school nurses, and unlicensed assistive personnel (UAPs). Participants were eligible if they were 18 years or older and worked in the schools during the COVID-19 pandemic and were willing to provide consent to participate in the study. The study-related information, including investigators’ contact information and a description of the purpose of the study, was sent via email to the district school nurse and school principals of each school district. The district school nurse solicited health staff at all schools in their district about the study which occurred during a mandatory training meeting for staff, but participation in the focus group was optional. Staff participation in the focus group discussion was not required by the district and all participants provided their informed consent. If eligible, participants were included in the study.

### Data collection

Data collection consisted of a demographic survey of de-identified baseline characteristics of participants and FGDs. The demographic survey was emailed to the study participants by their district lead nurse before the FGDs, and participants were allowed to submit over email or submit them in person before the FGD. We conducted two FGDs using a discussion guide consisting of ten open-ended questions (available via supplemental materials). One FGD was conducted in-person with 23 participants at an outdoor location and the other FGD was conducted over Zoom with 25 participants. Because the focus groups were conducted during the pandemic, and when social distancing measures were being observed, the research team worked with the school districts to conduct the FGDs in a manner that was acceptable to their district and convenient for their participation. This resulted in a larger focus group size than recommended to reduce the burden on the districts for school health professionals to participate (i.e., utilizing school health staff to coordinate a single meeting time as opposed to multiple). The discussions were moderated by an investigator trained in qualitative research methods, and another investigator took notes during the discussion.

In the Zoom focus group, participants had the option of using video or not, but everyone had the opportunity to unmute and speak during the focus group. The recording feature in Zoom was used to record audio. All participants were encouraged to raise their hand to speak or use the chat feature. The moderator called on those with hands raised, and the chat was monitored by a second investigator who let the monitor know about chat comments/questions. In the outdoor focus group, all the participants were seated in a circle so that everyone was able to see each other. There were three recording devices in different areas to record audio and two note takers. Participants were asked to raise their hand to provide their views.

### Data analysis

The audio recording was transcribed verbatim by a research team member and compared with the notes. A code book was developed using both inductive and deductive coding. Deductive coding was used based on our previous media analysis of the experiences of school nurses during the COVID-19 pandemic [15]. Thematic content analysis was used to generate codes and themes (Table S1). The socioecological model (SEM) was used to analyze the coded data at different SEM levels: individual, interpersonal, community, organizational, and societal levels [21]. Two investigators coded the data independently. A third investigator confirmed that conclusions drawn were supported by both interpretations of the FGD transcripts. Data was analyzed using the Atlas.ti software version 22 [22], and key quotes per theme in each level were identified by one investigator and confirmed through consensus by a second investigator. A registered school nurse consultant with extensive experience in research was included in the research team to ensure accurate interpretation of results and communication of key discussion points.

## Results

### Demographic survey results

A total of 48 participants were included in the FGDs. Thirteen participants did not complete the demographic survey; therefore, demographic data are reported on 35 participants (Table 1). The greatest proportions of participants were 35–45 years old (14/35, 40%), female (35/35, 100%), and non-Hispanic White (25/35, 71.4%). The greatest proportion of participants had 3 to 5 years of work experience (9/35, 25.7%) and nearly a third had been in their current position for 1 to 3 years (11/35, 31.4%). Most participants were unlicensed assistive personnel (UAPs) (23/35, 65.7%), followed by licensed school nurses (registered nurses) (9/35, 25.7%), with 3/35 declining to answer. Forty-six percent of participants (16/35) reported a change in their salary after the pandemic. Slightly more than half of the school health staff reported not being involved in the district level weekly COVID-19 Zoom meetings (18/35, 51.4%) to plan policies for the school district. However, 14.3% (5/35) were always involved in these meetings and served on the frontline at the district level regarding COVID-19 school policies.

**Table 1:** Participant demographics, work experience, and involvement in COVID-19 calls

Characteristics			N/35 (%)
Gender	Female		35 (100)
Race	Black or African American		1 (2.9)
	American Indian / Alaska Native (AIAN)		3 (8.6)
	Non-Hispanic White		25 (71.4)
	Multi-race		4 (11.4)
	Decline to answer		2 (5.7)
Age	< 34 years old		4 (11.4)
	35–45 years old		14 (40)
	46–55 years old		11 (31.4)
	> 55 years old		5 (14.3)
	Decline to answer		1 (2.9)
School grade level	Elementary (PK-6)		14 (40)
	Middle (5–8)		5 (14.3)
	High School		3 (8.6)
	Multi-grades (PK-12)		13 (37.1)
Years of work experience	< 1 year		6 (17.1)
	1–3 years		7 (20)
	3–5 years		9 (25.7)
	5–10 years		4 (11.4)
	10–15 years		4 (11.4)
	> 15 years		4 (11.4)
	Declined to answer		1 (2.9)
Years of experience in the current position	< 1 year		7 (20)
	1–3 years		11 (31.4)
	3–5 years		8 (22.9)
	5–10 years		2 (5.7)
	10–15 years		3 (8.6)
	> 15 years		3 (8.6)
	Declined to answer		1 (2.9)
Licensed nurse	Yes		9 (25.7)
	No		23 (65.7)
	Declined to answer		3 (8.6)
Salary change	Yes		16 (45.7)
	No		12 (34.3)
	Declined to answer		7 (20)
Type of salary change	Increased		16 (45.7)
	Decreased		1 (2.9)
	Stayed the same		5 (14.3)
	Declined to answer		13 (37.1)
Participated in COVID calls	No		18 (51.4)
	Yes	Occasionally	3 (8.6)
		Sometimes	0
		Often	7 (20)
		Always	5 (14.3)
	Declined to answer		2 (5.7)

### SEM thematic analysis

**Fig. 1:**
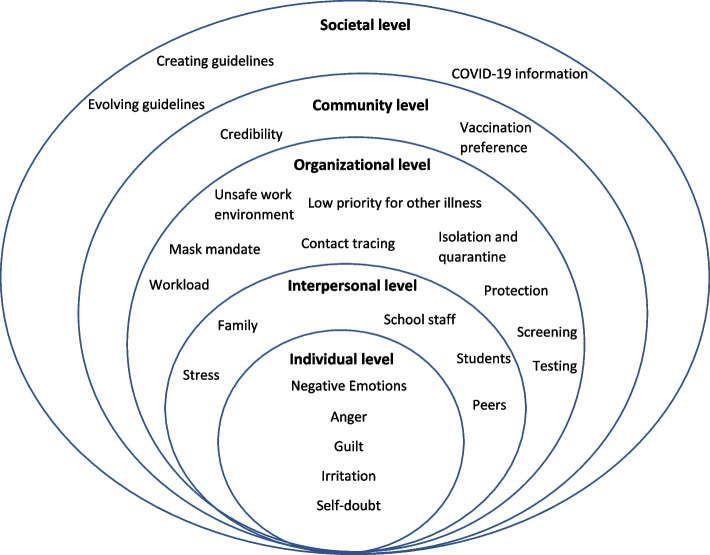
Socioecological model of COVID-19 experiences among school health staff in Arizona. Individual level themes included negative emotions, with anger, guilt, irritation, and self-doubt being the major emotions that emerged. Interpersonal themes included school health staff’s interactions with other school staff, their families and those of students, students, peers, and subsequent stress from these interactions. Organizational level themes included new tasks contributing to workload, lack of safety in the work environment, impacts on school health staff’s ability to address non-COVID-19 illness, and interventions implemented in schools (e.g., mask mandates). Community level themes included credibility of school health professionals and vaccination preferences. Societal level themes included the creation and evolution of guidelines and COVID-19 information

At the individual level, the most prominent theme was negative emotion, including anger, guilt, irritation, and self-doubt. At the interpersonal level, themes emerged around stress due to interactions with colleagues, parents, and family. Other interpersonal experiences included offering support for students and families and educating parents and school administration. At the organizational level, a dominant theme was workload, including tasks such as contact tracing, managing isolation and quarantine, screening, and testing. Other organizational level themes included managing mask mandates, de-prioritization of core functions that are non-COVID-19 health concerns, and safety at work (views on risks from COVID-19). At a community level, school health staff reported experiencing increased professional credibility in their communities and feeling valued due to being prioritized for vaccinations. At a societal level, emergent themes included methods by which school health staff gained information and guidance for schools. They often had limited support in interpreting, developing, or implementing guidance, resulting in having to manage or develop guidance or adapt to constantly evolving guidance provided from outside entities. Depiction of these themes relative to each SEM level can be seen in Fig. 1.

#### Individual level

##### Themes 1–5: negative emotions, anger, guilt, irritation, self-doubt

A variety of negative emotions (i.e., anger, guilt, irritation, self-doubt) were experienced by participants due to changing COVID-19 guidelines, and pushback regarding implementation of this guidance from parents and colleagues, including teachers. One participant commented on the challenges in communicating with parents,

* “We're just following the guidelines that are pushed to us and- but then trying to extend it to the parent. And then again, our heart strings are pulled, and we absolutely just go home at the end of the day just exhausted, because we're like, well, how many lives could be ruined today?”*

##### Themes 3 and 5: guilt and self-doubt

Guilt and self-doubt were especially apparent, regarding both tasks at work and at home.

Participants commented on feeling they were not doing enough at work at home due to the sudden increase in the work demand, raising feelings of inadequacy and internal conflict:

*“I keep telling myself, I have to be better.”*

*“You always feel sort of torn as a parent, right? – that you're not doing enough on either side. But that was like, just so in my face. Like, I wasn't doing enough for my children, and I wasn't doing enough at work. And that’s just it – that was definitely the hardest, I think, I've ever, like, professionally had.”*

*“I feel like I'm just barely bobbing above water to get through the day. And because I'm always that one who's really right on top of it, and for me, that's the hardest part, is – just feeling like nothing [is] getting done that I need to get done”*

#### Interpersonal level

##### Theme 1: family

The participants reported experiencing stress at work due to colleagues, parents, and family, including the families of students. Calling parents and asking them to take their child home due to COVID-19 symptoms was one of the major stressors reported:

* “The biggest stressor was calling parents. Just never knew how the conversation would go and, more often than not, parents were rude and not happy, right?!”*

*“Some parents were understanding while others were not ones that made it very –Those were the ones that made it very clear how they felt. They resorted to threats and threatening behavior. They called us every name in the book, even racial comments. It caused my job to no longer be fun and fulfilling, and it took away the happy spark.”*

School health staff also endured emotional burdens related to angry parents and teachers when children became ill. Parents sometimes refused to pick up their child from school and stated they felt their child just had cold. School health staff were put in the difficult position of having to consider the safety of the school and handle the pushback from parents. Participants also mentioned that some parents were undergoing family and financial hardships, which presented difficulties with adherring to the school protocols.

*“Because those kids have had to, you know, stay home, but it makes absolutely no sense. And you hear the, you know, the tragic stories, and the dad whose wife is in rehab, you know, whatever. And he doesn’t have a car, and he’s got kids, and now they’re positive. But then they have to stay home.”*

Participants reflected on how the long working hours of the pandemic affected their own families:

*“Ain’t worth that nine-year relationship. I just didn’t have anything else to give, and it- it’s sad. We’re still best friends. We’ve been friends for 35 years, but he got sick of me not having enough.”*

*“It’s all overwhelming. You take it out on your family or your friends or your relationships. And I have another, you know, I mean another position where lots of people are leaving their jobs because it’s so difficult managing your own personal experiences alongside everybody else’s experiences.”*

##### Theme 2: stress

Participants used coping strategies and sought professional help to overcome the stress and challenges due to the pandemic, while others found support in administration and close colleagues:

* “I've had to deal with a lot of challenging leadership things, and I couldn't have done it without my therapist.”*

*“Thinking that it helped me cope by having the supportive administration, other office staff attendance –Like, we all had to on the fly, just work together and help these families.”*

##### Theme 3: students

School nurses also helped students who had anxiety and fear due to the pandemic. They mentioned that some students had lost family due to COVID-19 and sought support through the school clinics:

* “Even the kids that have the anxiety – they’re not quickly fragile, but they come to the health office because they’re not feel-, quote unquote, ‘feeling well,’ and they really do need just the time to talk.”*

##### Themes 1, 4, and 5: family, school staff, and peers

School health staff were also required to disseminate updated COVID-19 guidelines and protocols to parents and school administration. School staff reported facing anger and frustration from parents when sharing sometimes changing or controversial guidance. This exacerbated school health staffs’ feelings of exhaustion and self-doubt. Similar experiences also occurred in communication with school administrators. One participant described difficulty in communicating the 10-day isolation (for students who tested positive for COVID-19) guidance with their principal and pushback on the length of the isolation period:

* “I think another difficult thing I had a lot of – staff, my principal, every time I had to talk to him, ‘That doesn’t make sense, that doesn’t make sense.’ And, like, people cannot understand, even though I felt like it was pretty general. You count 10 days and there it is. ‘It doesn’t make sense, how is that 10 days?’”*

#### Organizational level

At an organizational level, there were changes in duties and new roles assumed by school health staff, including contact tracing, isolation and quarantine, enforcing the mask mandate, screening, testing, and vaccination. This sometimes resulted in lower priorities for providing care for other acute and chronic illnesses.

##### Themes 1 and 2: contact tracing and workload

School health staff reported that contact tracing was one of the most time consuming and exhausting tasks added to their workload, and this is generally not a process in which school health staff have had previous training. They had to work during non-working hours and weekends to inform the staff and parents before students came to school and were not paid for this work. They also had to inform the health department of positive children and staff and maintain the contact tracing records.

* “It's like having two full time jobs: working evenings, weekends, holidays. Any time an email comes in, you have to start the process of contact tracing, and gathering information for reports, all while trying to do our actual daily tasks. Lots of exhaustion.”*

*“We got a positive, would contact trace and contact each person, and then we emailed the district to inform the health department. Attendance was notified at the end of the day who all was being quarantined from the teachers.”*

##### Theme 3: isolation and quarantine

While isolation and quarantine were interventions happening on a societal level, school health staff were specifically engaged in implementing these interventions at the organizational level of schools. The COVID-19 protocols changed frequently, and one school nurse mentioned how different schools responded to isolation and quarantine measures. School health staff reported requesting school administration for an isolation room, but they had challenges, as the administrative staff did not understand the need at the beginning of the pandemic:

* “I mean, heck, even being asked at the very beginning of the of the pandemic time, you know, like, we need we need an isolation room.”*

This changed for some schools, however, over the course of the pandemic:

*“So, in the in the very beginning, there were a few schools that didn't want to have an isolation room. They didn't think they needed one. We came a long way, let me tell you. One school was very, very unique, and they had a big tent outside of the health office where they would isolate the students.”*

##### Theme 4: mask mandate

School health staff played a key role in mask mandate adherence among the students, school staff, and parents. They discussed the difficulties with implementing mask mitigation at the administrative level due to the political debate of mask mandate in the state of Arizona:

* “The great mask debate has been very ugly… It got really intense, especially at an administrative level.”*

Masks were available for students in most schools. However, school health staff faced angry parents. Some children wore masks while others did not, making school health staff concerned about exposure to COVID-19. Some students had mask exceptions for religious reasons. Other challenges of poor mask compliance included difficulties in getting elementary school children or children with special needs to adhere, and students having challenges with masks when involved in sports activities. Even during these difficult times, school health staff gave extra attention to the children with special needs to keep them on campus. As these children could not wear the mask for long times, they came up with an alternative strategy to provide them with face shields instead of face masks, despite face shields being less effective [23]:

*“The other thing I did want to bring up was being we are in an inclusion school district. So having all of our kids – not have [having] a separate classroom for our special needs kids, you know, we had to order the face shields, because they wouldn't wear the masks. Or we'd have to find all these enclosed spaces to house these kids to give them all the services that they needed to keep them on campus. So that was a big struggle to get through this whole past year and a half.”*

##### Theme 5: screening

In some schools, temperature checks before entering the school were mandatory. They were used for screening all students, and were time consuming, meaning school health staff had to arrive earlier than their usual time.

* “Temperature checks every morning as people walk through the door every single student, uhm good 3-4- or 500 kids. And that- that was- took up a lot of the morning, where if someone got hurt on the way to school or whatever, they, you know, have to have someone else take over or check temperatures standing out in the cold.”*

*“I would have to come here, sometimes earlier than my actual clock-in time, to do the temperature checks. Then I would get a call later. The kid on the bus who has a fever or is, you know, has some sort of symptoms.”*

##### Theme 6: testing

One school district was not involved in COVID-19 testing at schools; however, other school district staff conducted COVID-19 testing at their schools and at the district level. Some schools had to use a single testing site in a community room. The testing was done initially for students and staff and later made available to anyone outside of the district who wanted to get tested.

##### Theme 7: low priority for other illnesses

School health staff felt they were providing less attention to children with chronic conditions and that these children did not receive enough medical attention because the school health staff were overwhelmed with additional COVID-19 duties. They were also not able to keep up with their regular work of writing care plans for kids with diabetes, asthma, or cancer or conducting vision and hearing screening and vaccinations:

* “From a nursing standpoint, we're supposed to be writing care plans for the sick kids in our district at our schools, and the-, I mean, the nice thing about this group is the health assistants know what to do at the school. They don't need that care plan, but we are supposed to have that plan in place, and we've got some pretty fragile kids that it's like, OK, yeah, I can throw out a standard diabetic plan, but I'm not having time to, like, individualize it.”*

*“If you read our job description, like, 5 years ago, we don’t do any of it. That’s how I feel. I mean, I can’t do hearing/vision. I maybe squeeze in one or two a day, just to keep up a little bit. But I'm behind [on] -everything, I mean, I have so many incompliances -immunization on campus.”*

##### Theme 8: screening

Some school health staff felt unsafe working during the pandemic due to the shortage of staff, working around infected students, and having an unclean work environment. A participant mentioned that they lost one of their colleagues, and some had left their jobs, which added more workload with no additional resources or substitutions to those remaining in school health staff roles. Exposures to students with COVID-19 were a concern, especially when parents delayed picking up the students or when parents sent students to school with fevers:

* “You know I don’t want to be in that room, right? I mean, I put him on my last bed, close the curtain, put a mask on, I mean- But that was a little ridiculous sometimes, where I felt that kid was positive.”*

*“The other thing about kind of not feeling safe is that some of the parents would be like, ‘Yay, finally, I'm going back to work. You're finally going back to school. Take this Tylenol because they know they're going to check your temperature on the way in and, you know, stay at school as long as you can.’ – because they didn't want to stay home, and there were probably a few parents that did that, although we couldn't, like, prove it. But, you know, that’s the way they would get them to school some days, because they knew those temperature checks were going to be first thing.”*

Participants also felt that the hygiene in some schools was compromised:

*“They literally just dump my trash cans over and leave the same bag in there. So, like, that was, like, a big one. I don't feel like they cleaned at all. I'm not even sure if they still clean our isolation rooms after we have kids.”*

##### Theme 9: protection

While participants described feeling unsafe, there was mention of certain measures that did enhance the feeling of safety regarding exposure to COVID-19. The school staff felt protected with stop signs attached to their doors with Velcro and plexiglass at the school health office. In the beginning of the pandemic, the plexiglass and stop signs on doors increased feelings of safety for some, safe enough to remove their mask:

* “And as silly as it sounds at the beginning, I was thankful for the plexiglass. That later became an annoying nuisance, but initially that plexiglass on my desk was, like, I felt like I could take my mask off at my desk and still be safe…”*

However, it should be noted that plexiglass effectiveness is variable and highly dependent upon its placing relative to airflow [23, 24], while masks, depending up on the filtering material and fit when worn, are a reliable way to reduce exposure [23, 25].

#### Community level

##### Theme 1: credibility

Participants reported there was a positive shift in the way they were valued before and after the pandemic. They were the source of information for the school personnel, parents, and students throughout the pandemic, helping them build trust and respect in the community:

*“Well, we work together, but now they know who we are, and we built trust. And I don't think that would ever happen any other way, because being in there, in school nursing for 12 years before COVID happened, to see the shift in respect and, like, admiration has been- it feels validating, like, they finally sort of get it and see it, which is nice.”*

##### Theme 2: vaccination experience

School health staff reported they were prioritized as first line health care workers and received early vaccination. They felt valued as members of the community for the service they did during the pandemic, with one participant stating, *“We were grateful that we were prioritized.”*

#### Societal level

##### Theme 1: COVID-19 information

School health staff received COVID-19-related information initially from the Centers for Disease Control and Prevention (CDC) and the Arizona Department of Health Services (ADHS) websites, based on which they created school policies. They later received information from district-level authorities and through the Pima County Department of Health services, or a group of individuals designated by the school who were responsible for ensuring guidance was distributed to school personnel and procedures were being followed including mandatory reporting requirements. School staff worked closely with the district administration to reduce the spread of COVID-19:

*“We also had the district-level task force pretty early on as well. That started with senior staff, so it was kind of mix, like the administrators, and nurses, myself, just different people around the district. That was 12 of us, and I was the lead on that.”*

##### Theme 2: creating guidelines

School health staff were involved in creating new guidelines for the schools based on CDC recommendations, even when the district had not announced any COVID-19 measures in early 2020:

*“We just took that information, because there weren’t [sic] a lot of information, template and forms, we had to adapt it for the school environment. A lot these guidelines was [sic] meant for the general public, or business, or that sort of things, but there wasn’t a lot that came out for schools”*

##### Theme 3: managing evolving guidelines

COVID-19 guidelines and policies changed over time, and school staff were involved in managing school health policies and creating new guidelines in the face of this evolving guidance, sometimes disseminating this information over Zoom:

*“We had a lot of Zoom meetings to share the information, and lot of people asked questions. We would have breakout sessions. Facilitators would meet with each of their group to communicate the information.”*

Guidance was sometimes met with a lack of understanding regarding how guidance may be different for schools than other environments:

*“Being on the receiving end, and they are-, again, because there is so much information, right? So we’re giving them our guideline and our information, but they see on the news- or, ‘But my husband say[s] this,’ or, ‘The company I work for says that.’ Yeah, but that is not a school environment. It is different than the classroom. It is different for this environment and organizations [sic].”*

## Discussion

### Key findings

This study highlights the vital role of school health staff in interpreting, developing, communicating, and executing guidance during the COVID-19 pandemic and the influence of this role on all socioecological levels: societal, communal, organizational, interpersonal, and individual (Fig. 1). Use of a socioecological model demonstrated that the most notable impacts on school health professionals in our study were on the interpersonal and organizational levels. Changes in work experiences and responsibilities have resulted in strain on family life, personal stress, and conflict with colleagues and partners in implementing COVID-19 guidance. The pandemic response in school environments resulted in new and unfamiliar tasks for school health staff, including administering vaccinations, enforcing mask usage, isolating sick students, managing quarantine guidelines, conducing COVID-19 testing and contact tracing, and educating and informing parents and school staff. These new tasks were added to an already excessive workload and required skills in which school health staff had not been trained. Additionally, COVID-19 tasks meant less time for other core school health functions. Even with these new responsibilities, extra hours, and increased occupational risks, most school health staff saw no pay increase or hazard pay. However, additional staff were hired in some cases to support the additional duties and some staff were provided with overtime pay.

Organizational changes in the form of new responsibilities put additional weight on an already strained system of school health and came at the cost of lower prioritization of care for students’ other illnesses. The long-term implications of this gap in care for students have yet to be seen but will likely be high, due to the fact that over 40% of school-aged children have one or more chronic disease [26]. School health staff are now “playing catch up,” as they address these healthcare gaps and adjust to a post-pandemic reality in which more immunocompromised students return to in-person education. This is occurring during a simultaneous shortage in registered nurses and other school health staff which continues to worsen as the school health workforce ages due to increasing retirements and decreased applicants interested in school health careers [27].

### Generalizability

The involvement of school health staff in developing and discussing pandemic guidance with school districts reported in our study is consistent with other studies. In Illinois, school nurses collaborated with the local health departments and formed a school nurse task force to develop COVID-19 toolkits [28], and school nurses in New Mexico collaborated with local health departments to implement COVID-19 policies in school districts [29]. These new partnerships have elucidated the leadership and decision-making skills of school nurses.

Negative emotions described on an individual level (e.g., anger, guilt, irritation, self-doubt) and experiences of stress and mental health problems due to increased workload among our participants are consistent with the findings of the CDC’s school nurse survey, which reported moderate to severe depression and anxiety during the pandemic among prekindergarten through grade 12 school nurses across the U.S. [19]. In prior research, need for social support was found to be associated with significantly higher odds of probable major depression (MD), generalized anxiety disorder (GAD), PTSD, and alcohol use disorder (AUD) among the health care workers [30]. Our research provides further evidence of a need for mental health resources and stress management strategy training for school health staff during future pandemics or outbreaks.

### Strengths and limitations

One of the key strengths of this study was the long-lasting relationships of two of our research collaborators with this community of school health staff. They have been working with them for the last ten years and were involved in the district level COVID-19 planning meetings. The established trust with the participants instils confidence that participants were honest and forthcoming in sharing their experiences in this study.

While our study offers important insights into the perspectives of school health personnel in two large school districts, the demographics of our participants are not representative of school health staff across Arizona. According to the 2021 Arizona school nurses and health survey results, a majority of their participants were registered nurses, with > 5 years of work experience which is different from our study participant characteristics (65.7% unlicensed nurse, 25.7% with 3–5 years of experience, Table 1) [20]. However, the majority of participants in the state survey reported working in public schools and elementary level schools, consistent with our study participants [20]. Despite lack of similarities between our participant demographics and those of participants in the state survey, our results add to the body of knowledge regarding challenges specific to Arizona and across the U.S., providing explanations as to why some school health staff may have felt unprepared or unsupported during the pandemic.

Another limitation included having more than twenty participants in each FGDs, more than the typical number of focus group participants (i.e., 7–8 people). One of the FGDs in this study was held in-person, while the other was held online over Zoom. The attentiveness of the participants in the online FGD was questionable as some participants had their camera turned off. The spontaneity of the discussions could have been affected by participants’ external environments (e.g., others in the room or ambient noise) and internet connectivity. It is also possible that some participants may not have had adequate time to voice their perspectives. However, we encouraged the participants to use the chat option to address this potential issue. This challenge was common to similar projects during COVID-19 due to many working in or preferring virtual options over in-person.

Lastly, due to FGDs being held in large groups, supervisors, senior, and early career school health staff were in the same FGDs, which may have resulted in an underrepresentation of job-related hierarchical problems in the discussions. However, due to the high workload of school health personnel, the research team determined it to be more feasible to hold large FGDs as opposed to small ones that separated school health personnel by rank or experience.

## Conclusions

The school health system, already strained prior to the pandemic, endured new pressures during COVID-19 at the expense of school health staff’s mental health and family life and at the cost of reliable healthcare for students with health needs unrelated to COVID-19. The organizational level in the socioecological model captured important side effects of increased COVID-19 job responsibilities for school health professionals, including the inability to address these regular duties. This may have long-term consequences and even immediate effects as school health staff work to address these gaps.

It is evident from research on the school health staff experience during COVID-19 that increased support at organizational and community levels is necessary during pandemics or other health crisis situations. At the organizational level, basic health infrastructure is lacking in schools. Basic infrastructure should include at least one full-time licensed nurse in every school. Pandemic specific support is necessary such as increased funds for additional staff and hazard pay. Additional support is also needed at the governmental level, relating to the societal and community levels in the SEM framework. While the districts in our study received support in the form of weekly calls, convened by the local health department, in which experts were brought in to answer questions (e.g., epidemiologists) and there was a designated person to communicate with school health staff, policy decisions were still left to school health staff in cases where they could not contact health department personnel to make decisions (e.g., shutting down classrooms during outbreaks). More communication between departments of education and health departments on policies to support school health decision making would ensure consistent policies across schools and decrease decision making burdens on school health staff. Lastly, a prominent issue raised in our study and in others was the consuming demand of contact tracing. Scaling up deployable infectious disease units and designated resources for school health staff would protect their time for maintaining core functions and caring for students with chronic health conditions during future pandemics and outbreaks. Further research is needed to evaluate the generalizability of the findings in this study and whether these suggested strategies for increased support of school health infrastructure could be effective in improving school emergency preparedness on a large geographical scale.

### Supplementary Information

**Additional file 1:** **Table S1.** Identified themes, codes, and associated definitions.

## Data Availability

The datasets used and/or analyzed during the current study are available from the corresponding author on reasonable request and will not include any identifiable information.
