# Supplementary material for: School health systems under strain: an example of COVID-19 experiences & burnout among school health staff in Pima County, Arizona
Source: BMC Public Health. 2023 Aug 25;23:1626. doi: 10.1186/s12889-023-16532-8 (PMC10463464; doi:10.1186/s12889-023-16532-8)
Supplement: Supplementary file 1 — Additional file 1: Table S1. Identified themes, codes, and associated definitions. [file 12889_2023_16532_MOESM1_ESM.docx]

**SUPPLEMENTAL MATERIALS FOR**

**School Health Systems under Strain: An Example of COVID-19 Experiences & Burnout among School Health Staff in Pima County, Arizona**

Amanda M. Wilson, PhD, MS;^1*^ Priyanka Ravi, MDS;^2^ Nicole T. Pargas, RN^3^; Lynn B. Gerald, PhD, MSPH^4^, Ashley A. Lowe, PhD, MSPH;^5,2^

1. Department of Community, Environment & Policy, Mel & Enid Zuckerman College of Public Health, University of Arizona, Tucson, AZ, USA
2. Department of Health Promotion Sciences, Mel & Enid Zuckerman College of Public Health, University of Arizona, Tucson, AZ, USA
3. Early Learning Resource Center (ELRC), Marana Unified School District, Marana, AZ, USA
4. Population Health Sciences Program, Office of the Vice Chancellor for Health Affairs, University of Illinois at Chicago, Chicago, IL, USA
5. Asthma & Airway Disease Research Center, University of Arizona Health Sciences, Tucson, AZ, USA

*Corresponding author: amwilson2@arizona.edu; 1295 N. Martin Ave. A233 Tucson, Arizona 85721

**Table S1.** Identified themes, codes, and associated definitions

| **Themes** | **Codes** | **Definition** |
| --- | --- | --- |
| **COVID-19 Information** | Health Departments | Received health information from health departments |
|  | Superiors | Received health information from superiors |
| **COVID-19 communication** | Creating COVID-19 guidelines | Role of school nurses in creating COVID-19 guidelines for school |
|  | Educating parents | Role of school nurses in educating the parents about COVID-19 |
|  | Informing school staff | Role of school nurses in informing and educating the teachers, administration and other staff about COVID-19 |
|  | Managing evolving guidelines | Type of strategies did they use to manage the evolving guidelines |
| **Credibility** | | The credibility (recognition) school nurses received for working during COVID-19 |
| **Emotions** | Fear | Fear experienced by the school nurses due to COVID-19 related work pressure or stress |
|  | Guilt | Guilt experienced by the school nurses due to COVID-19 related work pressure or stress, feeling of not being very helpful to others |
|  | Irritation | Irritation experienced by the school nurses due to COVID-19 related work pressure or stress |
|  | Self-doubt | Self-doubt experienced by the school nurses due to COVID-19 related work pressure or stress. Doubting their work capability. |
|  | Stress | Stress in any form (physical, mental or others) experienced by the school nurses due to COVID-19 related work |
|  | Support | Support received by the school nurses during the pandemic |
| **Inequity** | | Experience of unfairness or injustice at work during the pandemic |
| **Parent hardship** | | The difficulties faced by parents during the pandemic |
| **Protection** | | Protection measures taken to prevent the school nurses from COVID-19 at work |
| **Safety** | | Safety measures taken to prevent COVID-19 at school |
| **Stress** | Colleagues | Reporting stress due to the colleagues from work (teachers, UAP, administration) |
|  | Family | Stress experienced due to family members |
|  | Parents | Stress experienced due to parents at school |
|  | Students | Stress experienced due to students |
|  | Work | Other stress experienced due to COVID-19 |
| **Student stress** | | COVID-19 related stress or anxiety experience by the students |
| **Unsafe workplace** | Sick kids | Feeling unsafe working with sick kids |
|  | Staff shortage | Feeling unsafe due to staff shortage |
|  | Unclean work environment | Having to do cleaning due to unclean work environment |
| **Vaccination experience** | | Experience of getting the COVID-19 vaccination  Mention of any vaccination by student or teacher or administering vaccination |
| **Workload** | Contact tracing | Strength and challenges faced by school nurses doing contact tracing |
|  | Isolation and quarantine | Strength and challenges faced by school nurses doing isolation and quarantining the children infected with COVID-19 |
|  | Low priority to other illness | School nurses mentioning the challenges in manage the illness other than COVID-19 |
|  | Mask | Strength and challenges faced by school nurses implementing the mask mandate |
|  | Screening | Strength and challenges faced by school nurses doing the screening for COVID-19 at the schools |
|  | Testing | Strength and challenges faced by school nurses involving in COVID-19 testing |

**Focus Group Discussion Guide: The School Nurse Perspective of COVID-19**

**SCRIPT:**

**Facilitator:** *Greetings [Good Morning/Afternoon]! I am [insert name] from the University of Arizona Mel and Enid Zuckerman College of Public Health and I work with a team of public health researchers who are interested in understanding the School Nurse experiences during the COVID-19 pandemic. We are interested in hearing more about your perspective with being a school nurse (whether licensed or unlicensed) during the pandemic or since March of 2020. The purpose of this interview is to allow you to share your voice regarding your experiences, feelings and challenges so that we can better understand the occupational health risks you experienced (e.g., pandemic-related fatigue, increased stress/anxiety or personal safety).*

*I am going to ask you several questions. I would like you to share as little or as much as you would like to share with me. This focus group is being recorded, but only so we can transcribe these interviews and re-examine your responses. All responses will be de-identified and aggregated into themes as to not personally identify you. I expect this interview to take approximately 90 minutes to complete. We ask that you are respectful of your colleagues and that you allow each person to have an opportunity to share their perspective, even if it is different from your own. There are no right or wrong answers, but we do appreciate your feedback and time with completing this group discussion.*

*Are there any questions that I can answer before we begin?*

**QUESTIONS:**

**Section A: COVID-19 Information & Evolving Guidelines**

**Facilitator:** *For the first few questions, I would like you all to think back to the beginning of the COVID-19 pandemic (i.e., March 2020).*

1. During the last school year (2020-2021 school year), where did you receive information about COVID-19?
   1. From who or what sources?
2. During the last school year (2020-2021 school year), how did you receive information about COVID-19 guidelines from the Centers for Disease Control (CDC) or Pima County Health Department?
3. How did you cope with evolving guidelines for implementing COVID-19 mitigation strategies at your school?
4. Before vaccines became widely available, what was your biggest challenge or stressor during that time period?

[*Ask group participants if they would like a small 5 minute break.*]

**Section B: Workload**

**Facilitator:** *For the next few questions, I would like you ask you about how your work load or job tasks changed as a result of the pandemic. I would like you to think back to March 2020 all the way to your current job tasks and how this has evolved over time.*

1. During the last school year (2020-2021 school year), in what ways did your job or workload change?
   1. What sort of new tasks did you take on after March 2020?
      1. Do you still do these tasks now?

**Facilitator:** *Let us talk a little bit about contact tracing in your school and how this related to your job.*

1. At your school, whose job was it to assist the health department with contact tracing or determine which children or staff were suspected cases, who was exposed, etc.?
   1. What was your role?
   2. How did you provide information to the health department?
   3. How did you provide information to parents/families?
   4. How did you provide information to teachers/staff?
2. How did your school handle isolation or quarantine procedures for students and staff?
   1. Were there any issues or problems with these procedures?
3. How did your school handle the screening of students and staff for COVID-19?
   1. How did you test students?
   2. How did you test staff?
4. What types of policies did your school have in place for wearing facemasks?
   1. What role did you play in implementing or monitoring facemasks on students or staff?

[*Ask group participants if they would like a small 5 minute break.*]

**Section C: Personal Safety & Stress**

**Facilitator:** *For the next few questions, I would like you ask you about how your work load or job tasks changed as a result of the pandemic. I would like you to think back to March 2020 all the way to your current job tasks and how this has evolved over time.*

1. What was your experience with obtaining the COVID-19 vaccination?
2. What things made you feel protected at school from COVID-19?
   1. Not protected?
3. Since March 2020, what sort of things made you feel stress or anxious while at school?
4. Were there ever any issues or difficulties with communicating with parents?
   1. Students?
   2. Colleagues?
   3. School administration?
5. Is there anything else that you would like to add?

**Facilitator:** *[Closing] It seems that our time together has ended and I wanted to take a minute to thank you for your time, your perspectives and authenticity. We appreciate all that you have done during this pandemic.*
